# Quantitative comparison of myocardial fiber structure between mice, rabbit, and sheep using diffusion tensor cardiovascular magnetic resonance

**DOI:** 10.1186/1532-429X-13-74

**Published:** 2011-11-25

**Authors:** Lindsey J Healy, Yi Jiang, Edward W Hsu

**Affiliations:** 1 Department of Bioengineering, University of Utah, Salt Lake City, Utah, USA; 2 Center for In Vivo Microscopy, Duke University Medical Center, Durham, North Carolina, USA

## Abstract

**Background:**

Accurate interpretations of cardiac functions require precise structural models of the myocardium, but the latter is not available always and for all species. Although scaling or substitution of myocardial fiber information from alternate species has been used in cardiac functional modeling, the validity of such practice has not been tested.

**Methods:**

Fixed mouse (n = 10), rabbit (n = 6), and sheep (n = 5) hearts underwent diffusion tensor imaging (DTI). The myocardial structures in terms of the left ventricular fiber orientation helix angle index were quantitatively compared between the mouse rabbit and sheep hearts.

**Results:**

The results show that significant fiber structural differences exist between any two of the three species. Specifically, the subepicardial fiber orientation, and the transmural range and linearity of fiber helix angles are significantly different between the mouse and either rabbit or sheep. Additionally, a significant difference was found between the transmural helix angle range between the rabbit and sheep. Across different circumferential regions of the heart, the fiber orientation was not found to be significantly different.

**Conclusions:**

The current study indicates that myocardial structural differences exist between different size hearts. An immediate implication of the present findings for myocardial structural or functional modeling studies is that caution must be exercised when extrapolating myocardial structures from one species to another.

## Background

Computational studies are increasingly used to help interpret empirical measurements or to investigate functions of the body beyond experimental limitations. Because structures of the myocardium such as the fiber orientation play a deterministic role in its material properties and functional behaviours, accurate simulations of cardiac functions require precise anatomical models of the myocardium. Anatomy-based models of the myocardium have been used in computational studies of both electrophysiology [1,2] and mechanics [3-5] of the heart. In electrophysiological studies, utilizing anisotropic fiber orientation information has led to improved predictions of the electrical activity in the heart [2,6]. Similarly, incorporation of fiber structure into mechanical models has helped better explain the structure-function relationships [7,8].

Despite the significance of the information, measuring myocardial fiber orientation can be difficult, the key challenges being the small size (notably for the mouse) and availability of specimens (for humans). Tissue structures including myocardial fiber orientations are conventionally measured using histology [9-13]. By characterizing the anisotropy of water diffusion exerted by the molecular environment, cardiovascular magnetic resonance (CMR) diffusion tensor imaging (DTI) [14] has emerged as a viable alternative with the advantages of being non-destructive, relatively convenient, and inherently 3D. Across the spectrum of species, the practical feasibility of DTI for characterizing the fixed mouse heart has been demonstrated [15]. In contrast, applications of DTI on the human heart are largely limited to *in vivo *studies [16-18], which, due to technical limitations, continue to have relatively low spatial resolution and measurement quality.

Two approaches have been undertaken to circumvent the difficulties of acquiring subject-specific, or at least species-specific, structural information for modeling. First, generalized approximations of the structures are used. The most common examples of this approach include the prolate spheroidal representation [11] and the linearly varying (as a function of transmural depth) myocardial fiber orientation of the left ventricular myocardium [19,20]. Second, when species-specific information is unavailable (e.g., in the cases of mouse and human hearts), the myocardial structure for the species is simply substituted by that from another [4,21]. The obvious drawback of either approach, especially the second one, is that its validity is essentially untested, due to the very same reason of scarcity that prompted its use. To date, although myocardial structures have been examined using DTI and other techniques in individual species, few studies have systematically and quantitatively investigated their variability across different species. The dissimilar methodologies (e.g., specimen preparation, scan parameters, myocardial regions and structural parameters examined) employed among different studies make direct comparisons difficult, even for a retrospective study. There has been one anecdotal report comparing a single human heart to a group average of canine hearts [22], which found that the human heart appeared more different than the canine hearts were from one another, though the statistical significance was undetermined.

The goals of the current study are to perform a systematic comparison of the myocardial structures, as characterized by DTI, among different species, and to test the validity of modeling the myocardium of one species using the known parameters from another. Because of the high number of animal species in existence, a comprehensive study of this kind is practically prohibitive. Instead, the study focuses on 3 species spanning the spectrum of the size of the heart, including the mouse (small), rabbit (medium), and sheep (large), which have all been used in both experimental and computational studies of cardiac functions.

## Methods

DTI datasets of fixed, intact normal mouse (male 129/ola strain, n = 10), rabbit (male New Zealand, n = 6) and sheep (castrated male Dorsett, n = 5) hearts were retrospectively obtained from unrelated studies [15,23,24] that were all approved by the appropriate Institutional Animal Care and Use Committees at the respective institutions (see referenced studies for details). Inclusion of all available data was considered more important than the unequal sample size, which can be accounted for through the statistical analysis. Rabbit and mouse hearts were flushed with saline and fixed in formalin immediately after excision. The sheep hearts were perfused with KCl prior to a saline flush. None of the hearts were fixed with left ventricular transmural stress, so their conformation approximately corresponded to either the end-systolic (for the mouse and rabbit hearts) or beginning-diastolic (sheep hearts) state.

**Figure 1:**
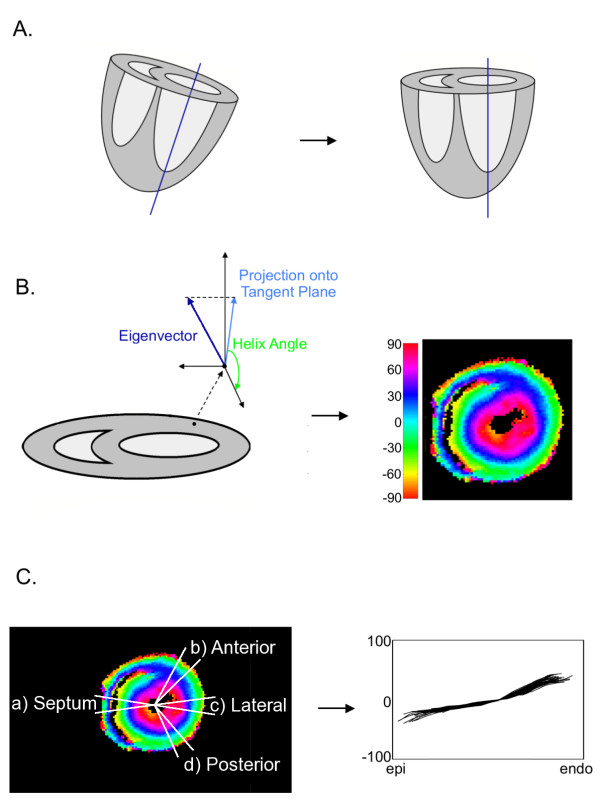
**Overview of image processing pipeline for data analysis**. Processing pipeline includes (a) realignment of images, (b) determination of fiber helix angle, and (c) sampling of helix angle.

Due to the different sizes of the heart specimens, MRI scans were conducted using different instrumentation (e.g., scanner field strength and RF coil size) and scan parameters (see Table 1). However, the DTI experiments were deemed comparable in terms of the encoding scheme (encoding directions and b factor), relative pixel size (with respect to the heart size), and SNR of the non-weighted (i.e., b0) scan. Each DTI dataset consisted of a single non-weighted and 12 diffusion-weighted 3D spin echo scans (128 × 128 × 128 matrix size) encoded in each of the same optimized set of 12 gradient directions. A schematic of the overall image-processing pipeline is pictured in Figure 1 and is described below. The diffusion-weighted scans were acquired and reconstructed using a novel "reduced encoding" DTI methodology [25], which combined partial k-space sampling and generalized-series reconstruction [26], and has been shown to offer significantly improved DTI acquisition-time efficiency (i.e., fiber orientation mapping accuracy versus scan time). The scan time for each DTI dataset was the same, approximately 9.1 hrs. Subsequently, diffusion tensors were estimated using a nonlinear least squares curve fitting on a voxel-by-voxel basis and diagonalized to determine the corresponding eigenvectors and eigenvalues.

**Table 1:** Anatomical, imaging and computed parameters for DTI scans on the mouse, rabbit, and sheep hearts.

	Mouse (n = 10)	Rabbit (n = 6)	Sheep (n = 5)
**Anatomical parameters**			
Apex-to-base length	6.7 ± 0.5 mm	19.5 ± 1.9 mm	76.6 ± 6.8 mm
LV wall thickness	1.5 ± 0.4 mm	3.9 ± 0.5 mm	12.5 ± 1.1 mm
**Imaging parameters**			
Scanner field strength	9.4 T	7.1 T	2 T
RF Coil diameter	1.4 cm	4 cm	10 cm
FOV	12.8 mm^3^	3.2 cm^3^	10 cm^3^
Pixel size	0.10 mm	0.25 mm	0.78 mm
Diffusion pulse width	5 ms	5 ms	10 ms
Diffusion pulse separation	7.5 ms	7.7 ms	15 ms
Maximum gradient	50 G/cm	40 G/cm	18 G/cm
Diffusion weighting b factor	1130 s/mm^2^	748 s/mm^2^	1175 s/mm^2^
**Computed parameters**			
LV wall thickness in pixels	15.6 ± 3.6	15.7 ± 2.0	16.0 ± 1.4
SNR of non-weighted scan	95 ± 15	102 ± 32	125 ± 34

When applicable, the entries are mean ± SD.

To obtain anatomically matching regions-of-interest (ROIs) for consistency of the analysis, the cardiac volumes were realigned via rigid body rotation using nearest neighbour interpolation so that the long axis of the left ventricle (LV) was approximately perpendicular to the image slices (i.e., the image slices corresponded to the true short-axis slices of the heart). The long axis was defined as the straight line connecting the apex of the heart through the approximate location of the tissue between the mitral and aortic valve as visually determined for each heart [12]. Myocardial structure represented by fiber helix angle was obtained in the mid-hemispheric region of each specimen where the LV was mostly cylindrical and can be most consistently and easily identified among the species. The myocardial fiber orientation helix angle was calculated as the inclination angle of the projection of the diffusion tensor principal eigenvector with respect to the circumferential imaging plane [10].

**Figure 2:**
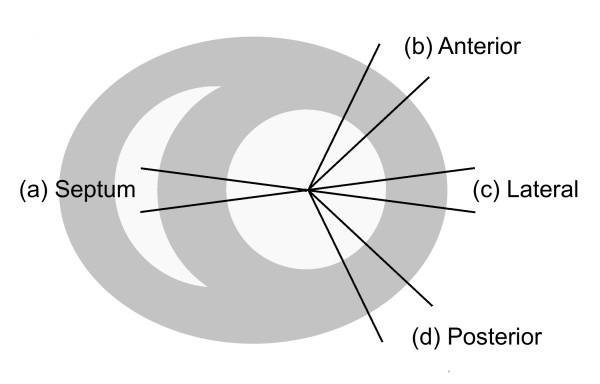
**Schematics of the ROIs for myocardial structural analysis**. ROIs include (a) mid septal wall, (b) anterior, (c) lateral, and (d) posterior free wall of the left ventricle. The same ROIs are defined for 3 consecutive image slices at the left ventricular equatorial plane, and myocardial structural parameters are sampled transmurally along radial paths separated by 1.5, for a total of 33 trajectories for each circumferential location for each heart.

Without loss of generality, the myocardial structural parameters of the left ventricle were investigated at 4 wedge-shaped ROIs, at (a) the middle of the septum, (b) the anterior free wall, (c) the lateral free wall, and (d) the posterior free wall, as schematically depicted in Figure 2. The anterior and posterior regions were placed midway between lateral free wall and the insertion points of the right ventricle. Within each wedge, the helix angles were sampled transmurally in 1.5-degree increments on 3 contiguous image slices for a total of 33 trajectories for each ROI in each heart.

Once the transmural fiber helix angles were sampled, each trajectory was processed for consistent demarcation of the epicardium and endocardium end-points. The epicardial end-point was taken as the minimum in the helix angle trajectory. The determination of the endocardial end-point was sometimes complicated by the presence of papillary muscles. Since in these instances the papillary muscle gave rise to a plateau in the helix angle trajectory, the beginning of the plateau was chosen as the endocardial cut-off. Following the definition of the two end-points, the helix angle trajectory was fitted to a straight line. The fitted y-intercept and r-squared value were used as the subepicardial fiber orientation and a metric of the linearity of the transmural rotation of the LV helix angle, respectively. Additionally, the range of the helix angles from the epicardium to the endocardium was computed to quantify the amount of transmural fiber rotation. The range was picked instead of the slope in order to eliminate the need for normalization based on wall thickness. The helix angle parameters for all trajectories in each ROI were then averaged for each animal.

Finally, to investigate the inter-species and inter-ROI variability of the myocardial structure, SAS™ software was used to fit a linear mixed model to the data and determine significance through the appropriate f or t-test using procedure MIXED (SAS Inst. Inc., Cary, NC). The different species and regions of interest were treated as fixed factors while the individual animal was treated as a random factor. The effect of the interactions between the species and region as well as the subepicardial helix angle, linearity of transmural rotation, and range of helix angle were tested. A p-value of 0.05 was considered significant.

## Results

**Figure 3:**
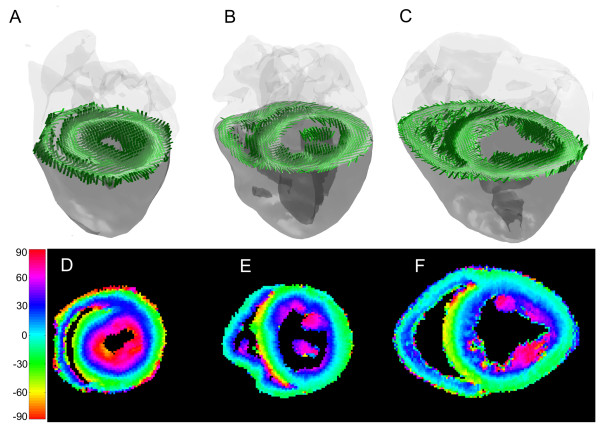
**Visualization of myocardial fiber orientation for representative mouse (a, d), rabbit (b, e) and sheep (c, f) heart specimens**. The myocardial fibers from the same short-axis slice are shown either as rendered cylindrical rods (a, b, c) inside semi-opaque volumes of the hearts viewed obliquely from an elevated perspective, or as falsecolor-coded helix angle maps.

Myocardial fiber orientations obtained in representative mouse, rabbit and sheep heart specimens are visualized in Figure 3, which consists of falsecolor-coded fiber orientation helix angle maps, and fiber orientations of the same short-axis slice rendered as cylindrical rods viewed obliquely from an elevated angle. Qualitatively, both methods visualization show the expected counter-clockwise transmural rotation of cardiac fibers from epicardium to endocardium. The fiber helix angles for the mouse appear to have a greater range than either that of the rabbit or the sheep.

**Figure 4:**
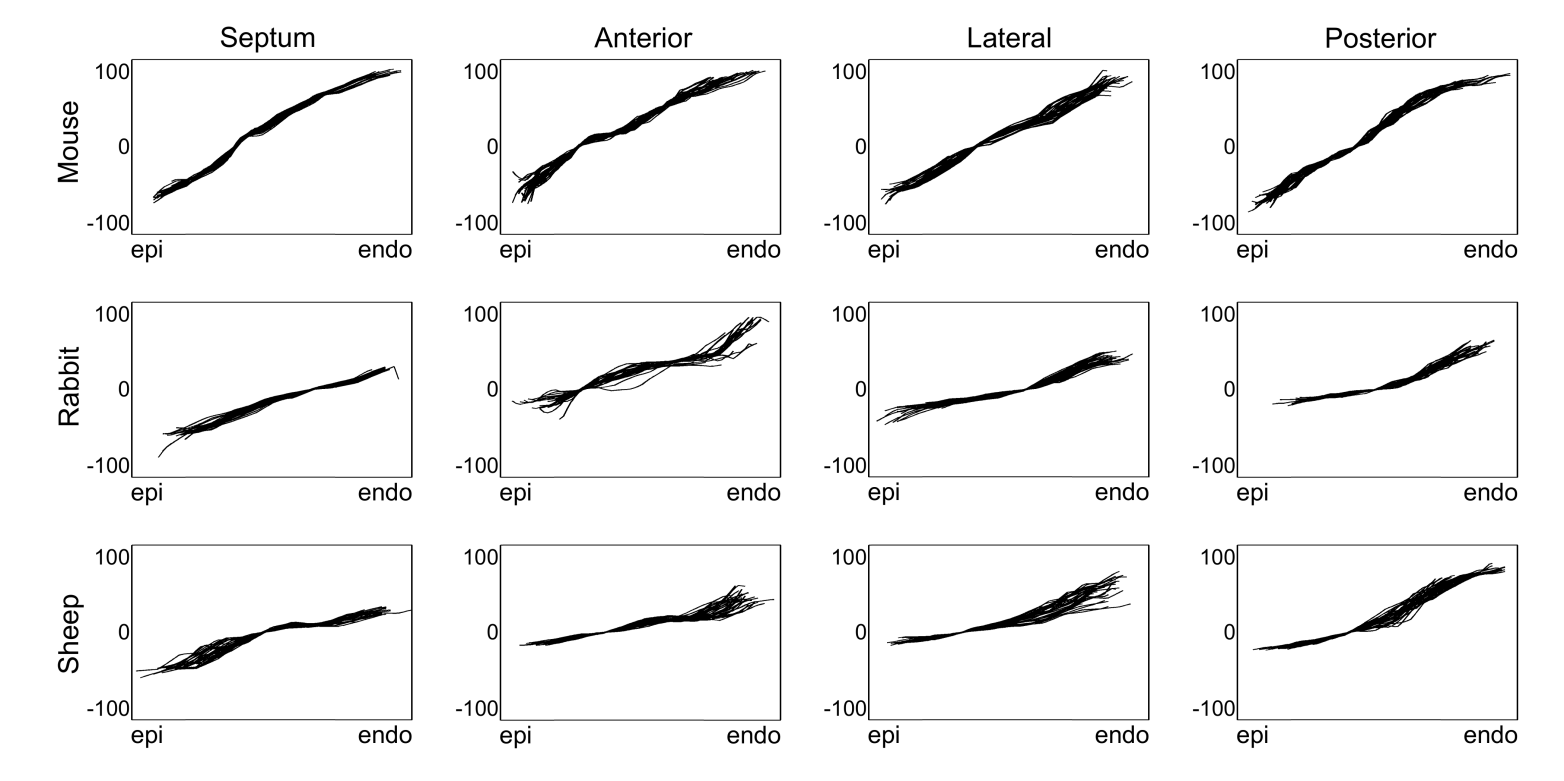
**Transmural trajectories of the myocardial fiber helix angle**. Myocardial fiber helix angles (in degrees) are shown as a function of circumferential ROI from representative mouse, rabbit, and sheep hearts. All individual trajectories are aligned at where the helix angle is zero to better show the slight variability in the ventricular wall thickness and the regional helix angle.

More precisely, representative transmural plots of the fiber orientation helix angle for the mouse, rabbit and sheep are shown in Figure 4. The helix angle trajectories for all species and zones also exhibit the expected counter-clockwise transmural rotation of the helix angle from epicardium to endocardium, confirming what is observed in the visualization of the helix angles in Figure 3. The trajectories are tightly clustered for each group, and within each zone for a given heart, indicating that the average for the wedge is an appropriate approach of measurement. Qualitatively, the subepicardial helix angle appears to be more negative in mouse than in either rabbit or sheep. Additionally, the transmural range the helix angle appears to be larger for the mouse than either the rabbit or sheep. In contrast, the behaviours of the transmural helix angle trajectories appear to be similar across different circumferential LV zones for all species.

The ROI-average values and standard deviations for the range, subepicardial helix angle, and linearity are tabulated in Table 2. The values for the helix angle range are similar to previously reported values for the mouse [15], rabbit [13], and sheep [27]. The transmural course of the helix angle appears to be more linear in the mouse than in the rabbit or sheep with a higher r-squared value in all zones for mouse than rabbit or sheep. Similarly, the range of helix angles is greater and the subepicardial helix angle is more negative in the mouse than either the rabbit or sheep. Results of the mixed factor analysis show that there are significant differences between mouse and sheep and mouse and rabbit (p < 0.01) for the subepicardial helix angle, range, and linearity of transmural helix angle, summarized in Table 3. Region did not have a significant effect on the range (p = 0.49) or linearity of fit (p = 0.27) but did on the subepicardial helix angle (p < 0.01). Specifically, the anterior free wall is different from the lateral free wall (p = 0.03) and the septal wall (p < 0.01) and the posterior free wall is significantly different from the septal wall (p < 0.01). Between the rabbit and sheep subepicardial helix angle and the transmural linearity were not significantly different (p = 0.34 and 0.39 respectively), however range of helix angle was found to be significant (p < 0.01).

**Table 2:** Measured myocardial fiber orientation.

	Species	Septal wall	Lateral free wall	Anterior free wall	Posterior free wall
Range	Mouse	129.8 ± 12.9	124.2 ± 18.8	127.7 ± 16.7	123.5 ± 17.9
	Rabbit	82.5 ± 17.9	75.8 ± 15.2	72.7 ± 19.1	78.7 ± 12.8
	Sheep	59.5 ± 16.1	58.2 ± 14.2	52.1 ± 14.3	58.1 ± 15.5
Subepicardial Helix Angle	Mouse	-58.6 ± 9.53	-50.2 ± 14.7	-35.9 ± 14.6	-46.9 ± 14.4
	Rabbit	-42.6 ± 14.9	-29.9 ± 14.6	-27.7 ± 11.4	-23.7 ± 13.9
	Sheep	-28.2 ± 19.2	-30.2 ± 8.74	-24.3 ± 9.64	-26.6 ± 9.26
Linearity	Mouse	0.98 ± 0.011	0.99 ± 0.011	0.97 ± 0.025	0.98 ± 0.017
	Rabbit	0.93 ± 0.14	0.94 ± 0.038	0.94 ± 0.050	0.97 ± 0.028
	Sheep	0.91 ± 0.070	0.95 ± 0.044	0.95 ± 0.055	0.94 ± 0.052

Fiber orientation is as characterized by the transmural range and linearity of the fiber helix angle, and subepicardial helix angle, as functions of both species and circumferential location of the ROI. Both the angular range and subepicardial angle are reported in degrees, whereas the linearity is dimensionless. All entries are mean ± SD.

**Table 3:** Summary of mixed analysis comparison of myocardial fiber orientation helix angle parameters in Table 2.

Comparison pairing	Mouse/Sheep	Mouse/Rabbit	Rabbit/Sheep
Range	Yes	Yes	Yes
Subepicardial Helix Angle	Yes	Yes	No
Linearity	Yes	Yes	No

"Yes" entries indicate that a significant difference was found for the comparison for the measure indicated.

## Discussion

Comparisons of the fiber helix angles indicate that there are significant myocardial fiber structural differences between any given two of the three species included in the current study. Specifically, the range of helix angles is larger, the subepicardial helix angle is significantly more negative, and the transmural course of the helix angle is more linear in mouse than in rabbit or sheep. Additionally, the range of the helix angles is significantly different between the rabbit and sheep, although neither the subepicardial angle nor the transmural linearity was significantly different between the two species. These findings strongly suggest that there exists species-dependent variability the myocardial fiber structure, at least in the LV as a function of animal size. This species-dependent variability would suggest differences are likely between human and animal myocardial fiber structure as well. An immediate implication of the finding is that cross-species substitution of myocardial fiber structural models is not a valid modeling practice.

The exact functional implications of the fiber structural differences among species are currently unclear, and are an obvious direction for future investigation. Based on the known structure-function relationships of the myocardium, at a minimum, one likely biomechanical impact of differing fiber structure is altered ventricular torsion. Since the transmural rotation of the myocardial fiber orientation is linked to the twisting motion of the heart [28], it is reasonable to predict that greater ventricular torsion would be associated with the larger transmural range of fiber helix angle found in smaller hearts. The increased torsion, in turn, would help compensate for the fewer myocytes spanning the ventricular thickness in maintaining the contractile efficiency. Indeed, a recent study on wall motion [29] indicated that there were significant variations among mouse, rat, and human hearts, specifically that latter had smaller torsion than either of the former two. Since human hearts are closer in size to sheep hearts, a possible explanation for the difference could be the size-dependent difference of the fiber structure.

One possible limitation of the current study exists in the different preparation of the fixed heart specimens, that the sheep hearts were perfused with KCl and thus fixed in the beginning-diastole state. In a previous study [30], it was determined that there was no significant difference between hearts fixed in an end diastolic and beginning systolic state but there was between the end diastolic and end systolic states. The differences in the myocardial fiber angles between different cardiac states were mainly attributed to the presence or absence of intraventricular volume. Consequently, because all heart specimens were fixed without intraventricular volume (or transmural stress) in the present study, perfusion with KCl is unlikely the cause of the different myocardial fiber structures observed between the sheep and either rabbit or mouse hearts.

A second potential limitation of the present study may exist in the inadvertent misalignment (e.g., in the determination of the cardiac long axis) of the image data, which would introduce systematic errors in the myocardial fiber angular measurements. To investigate the degree to which such misalignment could impact the current measurements, the image data of selected hearts were deliberately tilted in the left-right or anterior-posterior axis by 10°, and the angular measurements were repeated in all circumferential regions of the hearts. The results (not shown) indicate that the deliberate misalignment introduced statistically insignificant differences in all measurements, including the transmural range and linearity of the helix angle. One the one hand, the error in determining the cardiac long axis was estimated to be less than 10°. On the other hand, in the extreme case that the misalignment occurs in either the left-right or anterior-posterior axis, which would cause maximum systematic errors in angular measurements in the regions along the same axis, the effects were found to be insignificant. Therefore, the myocardial fiber structural differences observed in the current study are unlikely a result of errors in the alignment of the data.

Finally, it is worth noting that the methodology of data analysis employed in the current study relied on manual registration and ROI-based comparisons, which are commonly used in both DTI [23,31,32] and non-DTI [10,33] studies of myocardial fiber structures. Advances in computational anatomy have made it possible to achieve more precise image data registration and perform voxel-based analysis via techniques such as large deformation diffeomorphic metric mapping (LDDMM) [34]. Moreover, besides the fiber structure, the myocardial laminar or sheet structure [12] has been a subject of recent DTI studies [35-37]. These areas of investigation, including their technical challenges (e.g., increased likelihood of false positive differences when performing a large number of comparisons), are beyond but not precluded by the scope and findings of the current study. Conversely, studies either using more sophisticated data analysis techniques or examining more dimensionalities of the data likely will uncover details that only underscore the present findings, that significant differences exist in the myocardial structures among different species.

## Conclusions

In summary, significant differences in the myocardial fiber structure as represented by the fiber helix angle were observed between any two of the three species investigated in the current study. The range of the helix angle, subepicardial helix angle, and linearity of the transmural rotation of the helix angle through the left ventricle were all significantly different between the mouse and rabbit and between mouse and sheep. Similarly, the transmural range of the helix angles was significantly different between the rabbit and the sheep. Although how these findings can be generalized to species not included in the current study is unclear, there is indication that myocardial structural differences exist between different size hearts. An immediate implication of the present findings for myocardial structural or functional modeling studies is that caution must be exercised when extrapolating myocardial structures from one species to another.

## Competing interests

The authors declare that they have no competing interests.

## Authors' contributions

LJH analyzed and interpreted the data and drafted the manuscript. YJ collected the data for this study. EWH participated in study design, interpretation of the data, and drafting the manuscript. All authors read and approved the final manuscript.
